# Hip Fractures: Clinical, Biomaterial and Biomechanical Insights into a Common Health Challenge

**DOI:** 10.3390/bioengineering12060580

**Published:** 2025-05-28

**Authors:** Yunhua Luo

**Affiliations:** 1 Department of Mechanical Engineering, University of Manitoba, Winnipeg, MB R3T 5V6, Canada; yunhua.luo@umanitoba.ca; 2 Department of Biomedical Engineering, University of Manitoba, Winnipeg, MB R3T 5V6, Canada

**Keywords:** hip fracture, clinical risk factors, osteoporosis, diabetes type 2, bone composition, composition imbalance, fall biomechanics, fracture prevention strategies

## Abstract

Hip fractures represent a significant public health challenge, particularly among the elderly, due to their high incidence, morbidity, and mortality rates. This review provides a comprehensive understanding of hip fractures through clinical, biomaterial, and biomechanical perspectives. Clinically, we examined key risk factors, including age, bone mineral density, and the high prevalence of falls, which account for over 95% of hip fractures. However, current clinical tools, such as FRAX, have notable limitations in accurately assessing fracture risk in individuals due to their reliance on statistical models, the treatment of interdependent risk factors as independent, and the omission of key variables like diabetes. From a biomaterial perspective, we analyzed bone composition—specifically the balance of inorganic minerals, organic proteins, and water—and its role in determining bone strength and fracture susceptibility. Various risk factors ultimately influence this composition balance, thereby affecting bone strength. Therefore, accurately measuring bone composition may provide a more reliable assessment of hip fracture risk. Although emerging imaging technologies such as dual-energy CT and MRI show promise for in vivo assessments of bone composition, these techniques still face significant challenges and remain an active area of research. Biomechanically, we explored the forces generated during falls, noting that impact forces can vastly exceed normal physiological loads and may exploit the anisotropic properties of bone, leading to fractures even in healthy individuals with strong bones. This understanding emphasizes the critical role of fall prevention in reducing fracture risk and highlights the limitations of using fall-induced fracture incidence as a validation metric for clinical assessment tools. Lastly, we discuss preventive strategies, including passive measures like environmental modifications for individuals diagnosed with low bone strength and proactive measures such as muscle strengthening and cognitive training. While passive measures are necessary for immediate protection, proactive strategies are more effective in the long term by addressing underlying risk factors for falls and promoting sustained bone health. This interdisciplinary review underscores the need to integrate clinical, biomaterial, and biomechanical factors to improve diagnostic accuracy, prevention, and treatment strategies for hip fractures, ultimately advancing public health outcomes in aging populations.

## 1. Introduction

Hip fractures represent a significant and growing global public health challenge, standing out among other types of fractures due to their high incidence in aging populations and their severe impact on morbidity and healthcare systems [1,2,3]. The prevalence of hip fractures is primarily driven by osteoporosis [4,5,6], a common bone disease among the elderly characterized by low bone mineral density (BMD). This condition weakens bones, making them more fragile and highly susceptible to fractures. In Canada alone, around 30,000 hip fractures occur annually [7], with the International Osteoporosis Foundation projecting that this number will rise significantly due to the aging population [1]. Globally, the burden of hip fractures is rapidly rising. In 2000, approximately 1.6 million hip fractures occurred worldwide [8], and by 2010, this number had surged to 2.7 million [9]. Projections suggest that by 2050, the hip fracture incidence may triple, particularly in regions experiencing rapid population aging, such as Asia and Europe [1]. The highest rates of hip fractures are observed in Scandinavian countries and North America, where incidences in elderly populations reach around 300–400 per 100,000 people [10]. In terms of sex, women are disproportionately affected, with nearly 75% of hip fractures occurring in older women [11]. Men account for about 25% of hip fractures; although the incidence rate is lower, the post-fracture mortality risk tends to be higher in men than in women [12].

Hip fractures have far-reaching consequences that extend well beyond the immediate injury, affecting not only individual patients but also their families and society at large. Mortality rates in the year following a hip fracture remain high, ranging between 10–24%, with some studies indicating up to 30% in certain populations [12,13]. In Canada, approximately 20% of hip fracture patients die within the first year [7]. For those who survive, less than half regain their pre-fracture level of independence, and around 40% are unable to walk without assistance [14]. This loss of mobility significantly diminishes quality of life, often leading to long-term disability, depression, and decreased social participation. From a socioeconomic perspective, the impact of hip fractures on healthcare systems is profound. In Canada, the cost of treating a single hip fracture can exceed $125,000 per patient when considering hospitalization, surgery, rehabilitation, and long-term care [15]. This is compounded by the fact that many patients never return to their prior level of functional independence, necessitating additional healthcare services and increasing the demand for rehabilitation facilities. Globally, the rise in hip fractures, driven by aging populations, is contributing to an escalating burden on healthcare resources. It is estimated that by 2050, the annual number of hip fractures will surpass 6 million worldwide [1], further straining healthcare systems that are already grappling with the costs of managing chronic conditions associated with aging. The societal impact is also significant. Families of hip fracture patients often face a sudden shift in responsibilities, with caregivers, often spouses or adult children, experiencing physical, emotional, and financial stress due to the demands of long-term care. The need for caregiving can also lead to disruptions in employment for family members, further contributing to the overall economic burden of hip fractures. This ripple effect emphasizes the importance of viewing hip fractures not just as a medical issue but as a broader public health and social challenge.

The current clinical approach for diagnosing hip fracture risk and treating pre-fracture conditions relies primarily on risk factors such as bone mineral density (BMD) and population-based statistical models. Although low BMD is widely regarded as a key biomarker for bone fractures, this approach has significant limitations. Studies indicate that approximately 50% of fractures occur in individuals with BMD values above the established threshold. BMD also serves as the primary target for many treatment options, particularly those aimed at osteoporosis; however, this focus may not be optimal, and may even be invalid, as BMD alone does not provide a comprehensive picture of bone quality and strength. While osteoporosis is a major contributor to hip and other fractures, it is not the only factor involved. Diabetes, especially type 2 diabetes, has also emerged as a significant contributor to fractures due to its rising prevalence [16]. Interestingly, diabetic patients often have higher BMD [17], contradicting the BMD-based criterion currently used for assessing fracture risk [18]. Therefore, relying solely on clinical risk factors such as low BMD may not fully capture the underlying mechanisms contributing to various fractures, underscoring the need for a multidisciplinary approach.

From a biomaterial and biomechanics perspective, bone fracture results from either insufficient bone strength, an excessively large applied force, or a combination of both. While clinical assessments typically focus on risk factors like low BMD and their indirect association with fracture risk, biomaterial and biomechanical insights address the direct contributors to bone strength and fall-induced impact forces. Recent advances have enhanced our understanding of these mechanisms, underscoring the crucial roles of bone composition, composition ratios, microstructure, and the anisotropic nature of bone strength in determining fracture susceptibility. Furthermore, studies on fall-induced impact forces and directional loading have shown that fractures can occur even in individuals with high BMD if the applied force aligns with a bone’s weaker orientation or if fall dynamics generate excessive impact forces. These insights suggest that fracture risk cannot be reliably predicted by piecing together clinical risk factors alone, as they do not account for the full range of mechanical and material variables involved. Despite the clear importance of these biomaterial and biomechanical factors, they remain largely unintegrated into clinical assessment and pre-fracture treatment strategies.

The objective of this comprehensive review is to piece together the puzzle of clinical, biomaterial, and biomechanical factors involved in hip fractures based on recent advances in the relevant fields, with the aim of improving fracture risk diagnosis and treatment for pre-fracture conditions. Integrating biomaterial and biomechanical insights into hip fracture mechanisms and clinical practice offers the potential to significantly enhance prevention and treatment strategies, ultimately reducing the incidence and impact of fractures across diverse populations. 

The remainder of this paper is organized as follows: Section 2, Section 3 and Section 4 examine the clinical, biomaterial, and biomechanical aspects of hip fractures, respectively. Section 5 discusses passive and proactive prevention strategies informed by this comprehensive understanding of hip fractures. Finally, Section 6 concludes with recommendations for future research. It should be noted that the references cited in this review were selected based on their representativeness and alignment with recent advancements. Consequently, many relevant papers are not cited, given the substantial volume of literature available on this topic.

## 2. Clinical Insights into Hip Fractures

Clinicians have identified risk factors associated with hip and other fractures primarily through epidemiological studies, retrospective data analyses, and longitudinal cohort studies, which allow for the observation of associations between specific characteristics such as bone mineral density (BMD); demographics; and health, lifestyle, and environmental data and fracture outcomes over time. Large-scale population-based studies have been instrumental in identifying fracture risk factors. Researchers analyze characteristic data from diverse populations, examining which characteristics correlate with higher fracture rates. Examples include the Framingham Osteoporosis Study and the Study of Osteoporotic Fractures (SOF), which have provided substantial insights into risk factors like age, sex, prior fractures, and comorbidities [4]. Retrospective data analyses, such as case–control studies and database reviews, have also been essential in identifying fracture risk factors. For instance, case–control studies like the Global Longitudinal Study of Osteoporosis in Women (GLOW) analyze risk factors by comparing individuals with fractures (cases) to those without fractures (controls), identifying prior exposures or characteristics that may have contributed to fracture risk [19]. Additionally, administrative database studies, such as those using the Medicare and Kaiser Permanente databases [20], allow researchers to examine historical data on large populations, tracking variables like bone mineral density (BMD), medication use, and health conditions. These databases facilitate broad-scale analyses and have been instrumental in identifying comorbidities, like diabetes and cardiovascular disease, that contribute to fracture risk. Longitudinal cohort studies, which follow groups of individuals over extended periods, offer complementary insights by tracking the progression of risk factors and fracture outcomes over time. The Women’s Health Initiative (WHI) [21] and the Osteoporotic Fractures in Men (MrOS) study [22] are prominent examples, linking lifestyle factors and BMD with fractures and demonstrating how changes in these variables influence fracture risk.

The following subsections provide a structured review of these risk factors and their association with fracture risk, examine clinical tools and methodologies that utilize this information for fracture risk assessment, and discuss the inherent limitations in their accuracy and predictive reliability. Through this examination, this section aims to offer comprehensive clinical insights into hip fractures and the challenges linked to diagnosis and prevention based on clinical risk factors.

### 2.1. Clinical Risk Factors and Their Association with Major Fractures

Numerous risk factors have been identified as associated with hip and other types of fractures [23,24]. Low bone mineral density (BMD) is recognized as a primary risk factor. Clinically, BMD measured by Dual-Energy X-ray Absorptiometry (DEXA) serves as the gold standard for osteoporosis screening. BMD also functions as a biomarker for bone quality and is often used as a proxy for bone strength. Other risk factors can be broadly categorized into demographic, lifestyle and nutrition, bone health and medication, and environmental factors. Alternatively, these risk factors may be grouped more succinctly into internal factors, encompassing the first three categories, and external factors. In the subsequent paragraphs, the association of each category with hip fracture risk is examined, supported by evidence from literature. It is important to note that although clinical factors such as BMD and demographic variables are commonly used in fracture risk prediction, their normal ranges and associated risk ratios are highly population dependent. These values can vary significantly by age, sex, ethnicity, geographic region, and the methodologies used for measurement. Therefore, in this review, we discuss the associations between clinical risk factors and hip fracture risk in a qualitative rather than quantitative manner, as presenting generalized thresholds without the appropriate context may be misleading.

- *Demographic Factors*

Older age is one of the most significant risk factors for hip fractures, with the risk increasing dramatically in individuals over the age of 65. Studies consistently show that the incidence of hip fractures rises exponentially with advancing age, particularly in individuals over 80 years [1,3,23,25]. As people age, bone mineral density tends to decline, often leading to osteoporosis, a condition that weakens bones and makes them more fragile and susceptible to fractures [26,27]. Additionally, aging is associated with sarcopenia, the loss of muscle mass and strength, which impairs physical stability and increases the likelihood of falls [28,29]. These changes in muscle strength, combined with age-related declines in vision and balance, make older adults particularly prone to falls, the leading cause of hip fractures. Furthermore, the prevalence of chronic conditions such as arthritis, diabetes, and cardiovascular diseases rises with age [30,31], further contributing to fracture risk. Older adults are also more likely to be on multiple medications, some of which can negatively affect bone health (e.g., corticosteroids) or contribute to dizziness and fall risk (e.g., sedatives and antihypertensives) [32]. Taken together, these factors create a complex interplay of risks that significantly elevates the likelihood of hip fractures in the elderly population.

Older women are significantly more likely to experience hip fractures than men, primarily due to their higher rates of osteoporosis [3,33]. One key factor is the sharp decline in estrogen levels after menopause [34,35]. Estrogen plays a critical role in maintaining bone density, and its reduction accelerates bone loss, which leads to a greater risk of developing osteoporosis. Additionally, women generally achieve a lower peak bone mass compared to men [36], meaning they begin with lower bone density, making the effects of post-menopausal bone loss more severe. Moreover, women have a higher proportion of trabecular (spongy) bone [37,38], which is more metabolically active and more prone to rapid turnover and bone loss compared to cortical (dense) bone, which is found in higher proportions in men.

- *Bone Health and Medical Conditions*

Bone health and medical conditions significantly influence hip fracture risk, as both directly affect the strength, structure, and functionality of bones, along with overall physical stability and susceptibility to falls [39]. Several bone diseases can weaken bone strength, thereby increasing the risk of hip fractures [40,41]. Among these, osteoporosis is the most prevalent in the elderly, significantly weakening bones, particularly the cortical bone, which is critical for supporting body weight [26,33]. Osteoporosis causes an imbalance in bone remodeling, where bone resorption exceeds bone formation, leading to decreased bone density. This process is driven by various factors, including aging, hormonal changes, genetics, nutritional deficiencies, and lifestyle factors. Osteomalacia is a condition in which bones become soft due to impaired bone mineralization, typically caused by a deficiency in vitamin D or phosphate [42,43]. This softening weakens the bones, making them more susceptible to fractures, including hip fractures. Similarly, Paget’s disease disrupts the normal bone remodeling process, leading to abnormal, enlarged, and weakened bones [44,45]. The rapid and disorganized turnover in Paget’s disease results in structurally compromised bones that are more prone to fractures. Hyperparathyroidism, characterized by excessive production of parathyroid hormone (PTH), leads to elevated calcium levels in the blood and increased bone resorption [46]. This excessive breakdown of bone tissue reduces bone density and weakens bones. Osteogenesis imperfecta (OI), commonly referred to as “brittle bone disease”, is a genetic disorder affecting collagen production [47,48]. This results in brittle bones that fracture easily, even with minimal trauma. Rheumatoid arthritis, an autoimmune disease, causes chronic inflammation in the joints and promotes bone erosion over time [49]. The disease also stimulates the production of cytokines [50], which accelerate bone resorption, further weakening the bone structure and increasing the likelihood of fractures.

In addition to bone diseases, other medical conditions can significantly influence hip fracture risk, as the human body functions as an interconnected system. For example, in individuals with chronic kidney disease (CKD), imbalances in calcium, phosphate, and vitamin D metabolism lead to abnormal bone mineralization and turnover [51,52], ultimately weakening bones. In people with diabetes, particularly type 2, bone mineral density (BMD) is often normal or higher, but bone quality is compromised [53,54]. This paradox occurs because high blood sugar (hyperglycemia) and insulin resistance disrupt osteoblast function and increase the accumulation of advanced glycation end-products (AGEs) in bone collagen, making bones more brittle and prone to fractures [53]. Similarly, cardiovascular diseases (CVD), such as atherosclerosis and heart failure, negatively impact bone health by reducing blood flow to bones, impairing their ability to regenerate and remodel. Poor circulation in these conditions can lead to weakened bones and a higher risk of fractures [55,56].

Several medications have been shown to influence hip fracture risk, particularly through their effects on bone health and body balance. Long-term use of corticosteroids is strongly linked to reduced bone mineral density and an elevated risk of fractures, as these medications increase bone resorption and decrease bone formation [57,58]. Antidepressants, especially tricyclic antidepressants and selective serotonin reuptake inhibitors (SSRIs), have been associated with a higher fracture risk [59,60], possibly due to their effects on balance, coordination, and fall risk, as well as their potential impact on bone metabolism. Additionally, anticonvulsants and certain proton pump inhibitors (PPIs) used for acid reflux have also been linked to decreased bone density and increased fracture risk when used long term [61,62].

- *Lifestyle and Nutrition*

Lifestyle and nutrition play a critical role in maintaining bone health and muscle strength [63]. Physical inactivity accelerates muscle loss (sarcopenia) and bone density reduction [64,65], which elevate the risk of falls and fractures, especially in older adults. Conversely, engaging in weight-bearing activities, such as walking, running, or resistance training, promotes bone remodeling, which helps maintain or increase bone density [66,67]. Regular physical activity also strengthens muscles, improving balance and reducing the risk of falls. Additionally, exercises that enhance balance and flexibility, such as yoga or tai chi [68,69], are particularly beneficial for older individuals by improving stability and coordination, thus lowering fall risk. Body weight plays a significant role in hip fracture risk, with body mass index (BMI) showing a U-shaped relationship to fracture risk [70,71]. Both underweight and obese individuals face elevated risks, but for different reasons. Being underweight is associated with reduced bone mass and less body fat [72], which decreases the natural cushioning during falls, thereby increasing the likelihood of fractures. Low BMI is often linked to insufficient bone mineral density, making bones more fragile and prone to breaking. On the other hand, obesity can impair mobility and balance, increasing the risk of falls that may lead to fractures [73,74]. Obesity is also paradoxically associated with poor bone quality, despite the fact that individuals with higher BMI tend to have higher bone density [75,76]. This discrepancy will be further explained in Section 3 from a biomaterial perspective. Certain lifestyle choices, such as chronic alcohol consumption and smoking, negatively affect bone health and strength [77,78]. Alcohol interferes with calcium absorption and disrupts bone remodeling, leading to lower bone density, while also impairing balance and coordination, making falls and fractures more likely. Similarly, smoking reduces bone mineral density by impairing the body’s ability to absorb calcium and increasing bone resorption, which contributes to higher risks of osteoporosis and fractures.

Proper nutrition is essential for maintaining bone health and reducing fracture risk. Adequate intake of calcium and vitamin D is crucial for bone mineralization and density [79,80]. Calcium is a fundamental component of bone, while vitamin D aids in its absorption. Deficiencies in these nutrients can lead to weakened bones and increase the risk of osteoporosis. Furthermore, sufficient protein intake supports both muscle and bone health [81,82,83]. Protein deficiencies can result in muscle atrophy, which compromises physical stability and increases the risk of falls.

- *Environmental Factors*

Environmental factors significantly influence fracture risk by affecting the likelihood of falls, which are the leading cause of fractures, particularly in older adults [84,85,86]. Hazards in daily surroundings—such as slippery floors, poor lighting, cluttered walkways, and uneven surfaces—heighten the risk of falls. Older individuals, who often experience reduced balance, vision, or mobility, are especially vulnerable to these environmental hazards. The availability or absence of safety features in homes and public spaces also plays a crucial role. Handrails, grab bars in bathrooms, and non-slip mats are essential in reducing fall risk. The design and layout of homes can affect the chances of falls [87,88]. Homes with stairs without handrails, high steps, or complicated layouts pose significant risks to elderly individuals with compromised balance or mobility. People living in multilevel homes are at a higher risk of falls compared to those in single-story homes [84,89], where fewer transitions between levels reduce the likelihood of accidents. Additionally, weather conditions significantly affect fall risk. Icy, wet, or snowy surfaces, common in colder climates, increase the chance of slipping outdoors, making falls more likely, especially for individuals with impaired mobility. Extreme temperatures can also affect balance and coordination, further elevating fall risk.

In general, environmental risk factors are highly diverse, unpredictable, and often beyond direct control, presenting significant challenges for preventive measures, particularly in contexts such as fall prevention and hip fracture risk.

### 2.2. Clinical Assessment Tools Derived from Risk Factors

Based on the association between risk factors and fracture outcomes, several clinical assessment tools have been developed to evaluate hip fracture risk, including Dual-Energy X-ray Absorptiometry (DEXA), FRAX, QFracture, and the Garvan Fracture Risk Calculator [90,91,92,93]. These tools primarily utilize statistical modeling of internal clinical risk factors, drawing from extensive population datasets and cohort studies. These tools differ in the number and types of risk factors they consider.

Though not a standalone risk assessment tool, DEXA is the gold standard for measuring bone mineral density (BMD) [91], a critical factor in assessing bone health and a major input in other risk assessment models like FRAX. FRAX is one of the most widely used tools globally to estimate the 10-year probability of hip fractures and other major osteoporotic fractures [93,94,95,96]. It incorporates several key risk factors, including age, gender, BMD at the femoral neck, prior fractures, parental history of hip fractures, smoking status, alcohol consumption, glucocorticoid use, and rheumatoid arthritis. Developed in the UK, QFracture is based on a large, prospective cohort study using data from millions of primary care patients [97]. It estimates fracture risk over a 10-year period and is widely used in the UK [98], though less so in other regions. QFracture considers factors such as age, gender, smoking and alcohol intake, history of falls, existing conditions (e.g., diabetes, heart disease), and medication use (e.g., corticosteroids) [99]. The Garvan Fracture Risk Calculator, developed in Australia, predicts fracture risk based on clinical factors and fall history. It is commonly used in clinical practice in Australia and parts of Europe [100,101], but is less widespread globally compared to FRAX.

### 2.3. Limitations and Challenges of Current Clinical Tools

DEXA is highly reliable for measuring BMD, which is considered the most important single predictor of osteoporosis and fracture risk. The reproducibility of DEXA measurements is excellent, with a coefficient of variation (CV) typically under 1% in clinical settings [102,103]. However, while DEXA is very accurate in assessing BMD, its ability to predict hip fractures independently is limited because it does not account for other crucial factors like fall risk [104], bone quality, and muscle strength. Studies show that about 50% of fractures occur in individuals who do not have osteoporotic BMD values [105], underscoring the limitations of DEXA when used alone. The predictive accuracy of FRAX has been reported as moderate [106,107], with Area Under the Receiver Operating Characteristic (ROC) Curve (AUC) values ranging from 0.70 to 0.75 for hip fracture prediction. The tool tends to underestimate fracture risk in certain populations, such as those with frequent falls or advanced age, since their fall risk is not fully incorporated [95,108,109]. QFracture has demonstrated performance comparable to FRAX, with reported AUC values ranging from 0.74 to 0.77 for hip fracture prediction [110,111]. QFracture appears to have better predictive ability for individuals with multiple comorbidities and a history of falls, making it particularly useful in elderly populations [99]. However, the accuracy of QFracture may be reduced outside the UK, as the tool is primarily validated for UK populations and may not include the relevant risk factors for different regions [107,111]. The Garvan Fracture Risk Calculator has demonstrated slightly higher accuracy in predicting fractures, with reported Area Under the Curve (AUC) values ranging from 0.71 to 0.76 [100,111]. Unlike FRAX, Garvan incorporates fall history into its model, which improves its predictive accuracy for those at higher fall risk. The studies indicate a significant need for improving the accuracy of these clinical tools. Despite ongoing efforts, advancements in enhancing their predictive reliability have been minimal.

The primary limitation of the current tools lies in their reliance on statistical modeling. These tools predict fracture risk by identifying broad population-level trends and applying them to individual cases [111]. While this approach works well for estimating average risk across groups, it often fails to account for the variability and complexities of individual patient profiles [112]. For example, these models provide probabilistic estimates based on aggregate data, which can miss specific nuances in a patient’s lifestyle, behaviors, or fall history [90]. As a result, these models may offer general insights but struggle to make precise, individualized predictions.

Many crucial risk factors are inherently difficult to quantify, leading to oversimplifications in risk models. Factors such as muscle strength [113], balance, and fall history significantly affect fracture risk [90], but are challenging to measure consistently and objectively [23]. Fall risk depends on a range of factors, including environmental hazards [24], medication use, and cognitive impairments [114], all of which can vary widely between individuals. Current models often rely on binary inputs (e.g., fall/no fall) or self-reported data, which do not fully capture the complexity of these factors, thus reducing the predictive accuracy of the tools.

Another limitation arises from the interdependence of clinical risk factors [115]. For example, bone mineral density is closely related to age, gender, and lifestyle factors such as smoking and alcohol consumption [116,117,118]. These interrelationships can introduce multicollinearity into statistical models [119], where the combined effects of multiple risk factors are not simply additive but overlapping. Without accounting for these correlations, the models may overestimate or underestimate the contribution of individual factors.

There also exist independent risk factors that current clinical assessment tools either underrepresent or entirely omit. Factors like environmental hazards (e.g., slippery floors, poor lighting) [84], cognitive impairments (e.g., dementia) [120], and the use of certain medications (e.g., sedatives, antidepressants) [121] can greatly influence fall risk and fracture outcomes. However, these variables are difficult to systematically incorporate into risk models due to their highly individualized nature and the complexity of their effects on fracture risk. As a result, clinical tools may miss key risk factors that could substantially alter a patient’s true fracture risk profile.

In conclusion, while current clinical assessment tools provide useful population-level estimates of fracture risk, their limitations highlight the need for more patient-specific biomechanical models. Such models would integrate detailed patient data, including bone quality, muscle strength, and individual biomechanical factors, providing a more accurate and personalized assessment of fracture risk. Emerging integrative approaches are advancing hip fracture risk prediction by combining patient-specific biomechanical modeling with Dual-Energy X-ray Absorptiometry (DEXA)-based measurements. Recent studies have demonstrated that finite element (FE) models derived from DEXA scans can simulate patient-specific stress and strain distributions under fall-related loading, thereby providing improved fracture risk assessments. For instance, Luo et al. [122,123], Yang et al. [124], and Leslie et al. [125] showed that automated FE analysis of DEXA hip scans generated fracture risk indices significantly associated with prior hip fractures, independent of FRAX-based predictions. Similarly, Grassi et al. [126] demonstrated that DEXA-based 3D FE models outperformed BMD in predicting hip fractures in elderly women. These hybrid models exemplify how biomechanical parameters can enhance DEXA-based diagnostics and support more individualized risk stratification.

## 3. Biomaterial Perspectives on Bone Strength

**Figure 1:**
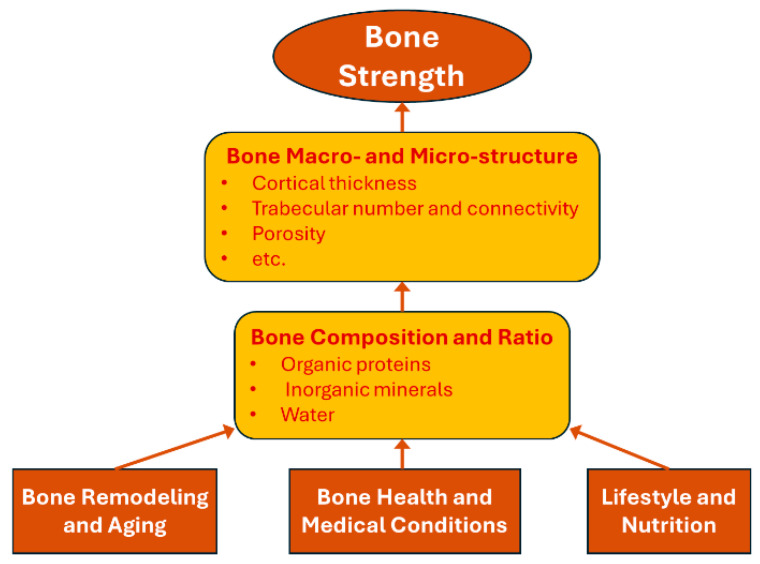
Factors affecting bone strength.

Hip fractures are primarily influenced by two biomechanical factors: bone strength and fall-induced impact force. This section focuses on the first factor, examining the biomaterial aspects that contribute to bone strength, while the analysis of fall-induced impact forces will be addressed in the next section. In the context of fall-related fractures, bone strength includes two critical properties: ultimate force, the maximum load bone can bear before failure, and toughness (or resilience), the bone’s ability to absorb energy before fracturing. As illustrated in Figure 1, bone strength is determined by its composition and structure, which are influenced by bone remodeling and aging, bone health and medical conditions, lifestyle and nutrition.

The following subsections will first examine the roles of bone composition and bone structure in regulating bone strength. Next, we will discuss how these factors are influenced by various clinical risk factors, including aging, bone health and medical conditions, and lifestyle and nutrition.

### 3.1. Bone Composition and Microstructure

From a material perspective, bone is a composite composed primarily of inorganic minerals, organic proteins, and water, each contributing uniquely to bone strength [127,128,129,130]. Organic proteins, predominantly collagen, form a flexible scaffold first, providing a structural matrix essential for subsequent mineralization [131]. This collagen framework, laid down by osteoblasts, serves as the foundation where inorganic minerals, primarily hydroxyapatite (a calcium phosphate compound), are deposited. This mineralized matrix strengthens the scaffold, imparting compressive strength and rigidity. Water, which makes up approximately 10–20% of bone mass, is distributed within both the mineral and organic phases, supporting tissue hydration, nutrient transport, and mechanical resilience under stress. This tripartite composition, i.e., minerals, proteins, and water, highlights bone’s nature as a composite material, where each component serves a specific mechanical function: mineral’s hardness and rigidity, collagen’s tensile strength and flexibility, and water’s viscoelastic properties for energy dissipation under loading. Understanding these components individually and as an integrated system is key to appreciating the material properties that contribute to bone strength and fracture resistance [127].

The ratio of these components—inorganic minerals, organic proteins, and water—varies significantly by anatomical site to meet specific physiological and mechanical demands [132,133,134]. For instance, cortical bone in the shafts of long bones, like the femur and tibia, has a higher mineral content than trabecular bone found in the vertebrae and at the ends of long bones [132]. This high mineral content in cortical bone imparts compressive strength and rigidity [135], essential for withstanding the weight-bearing loads typical in the lower limbs. In contrast, trabecular bone has a higher proportion of organic proteins and water, contributing to its flexibility and shock-absorption capacity [136]. This composition allows trabecular bones to dissipate energy effectively, which is crucial for joints exposed to multidirectional forces, such as the spine and the femoral neck. Additionally, collagen concentration is higher in areas where tensile strength and elasticity are essential for enduring repetitive stresses, such as the upper part of the femoral neck.

The quantity and quality of organic proteins, particularly collagen [137,138], are critical in determining bone strength [139]. High-quality collagen provides a robust and flexible scaffold that enhances tensile strength and resilience by supporting mineral attachment. Key factors in collagen quality include cross-linking patterns and fiber orientation; well-organized, tightly cross-linked collagen improves energy absorption and resistance to tensile forces, crucial for preventing fractures [140,141]. Conversely, deficiencies in collagen quantity or disordered cross-linking reduce bone’s ability to absorb mechanical energy, making it more brittle. While inorganic minerals and water are relatively abundant and easy for the body to acquire, the unique structural properties of collagen are more challenging to regenerate with age [142,143]. Proper hydration within this matrix helps distribute the load evenly, while dehydration or poor water retention weakens bone resilience. Recent computational studies have advanced our understanding of water diffusion dynamics within bone’s hierarchical structure, particularly at the nanoscale interface of collagen and mineral phases. Bini et al. [144] employed a 3D random walk model to demonstrate how variations in mineralization levels alter water diffusivity in collagen fibrils, with implications for mass transport and local mechanical properties relevant to fracture resistance. Similarly, Lemaire et al. [145] used molecular dynamics simulations to investigate the behavior of water at the collagen–mineral interface, revealing how hydration influences bone’s toughness and viscoelasticity. From a continuum perspective, Cowin’s poroelastic model [146] further formalizes how fluid flow within the porous bone matrix contributes to its mechanical response under loading. Together, these studies highlight the critical role of nanoscale water dynamics in bone quality, and support the integration of such mechanisms into future biomechanical models of fracture risk.

Bone microstructure is also a crucial determinant of bone strength [147,148], particularly through its contribution to anisotropy [149]—the property by which bone exhibits different strengths depending on the direction of the applied force. In long bones, such as the femur, cortical bone is strongest along the longitudinal axis, aligned with the primary load-bearing direction [150,151]. Tightly packed, parallel osteons allow cortical bones to withstand substantial loads, providing the rigidity necessary for supporting body weight and resisting bending and torsion. In contrast, trabecular bone displays anisotropic strength that depends on the orientation of its trabeculae, which aligns with the primary physiological forces experienced at specific sites. Studies have shown that the femoral neck exhibits significantly greater strength when forces are applied in a downward direction (from proximal to distal) compared to upward loading (from distal to proximal) [152,153]. This is because the superior region of the femoral neck, which is rich in cancellous bone, is better suited to resist tensile stresses generated by body weight, while the inferior region, composed predominantly of cortical bone, is optimized to bear compressive loads. However, during a fall, the direction of the impact force is often reversed [153], subjecting the bone to stress patterns that oppose its natural load-bearing design and increasing the risk of fracture [152]. This site-specific microstructure across anatomical regions balances strength with flexibility, as cortical bone provides stability where needed, while trabecular bone’s porous structure accommodates dynamic forces. These structural adaptations enable bones to meet various mechanical demands, enhancing resilience while minimizing weight.

### 3.2. Changes in Bone Composition and Quality During Remodeling and Aging

Bone remodeling is a continuous process in which osteoclasts resorb old bone and osteoblasts secrete osteoid, a collagen-rich organic matrix that serves as the scaffold for subsequent mineralization [154,155]. Collagen’s quality and quantity are critical to this process [156]. However, with aging, the body’s capacity to produce high-quality collagen declines substantially, weakening the organic matrix that serves as the foundation for mineralization [157]. As people age, their collagen quality declines [158], primarily due to changes in cross-linking patterns and fiber orientation. Abnormal cross-linking and fiber disorganization reduce collagen’s resilience and impact-absorbing abilities. Additionally, reduced collagen quantity limits the bone’s ability to regenerate a robust framework for mineral deposition. Aging also impacts water retention within the collagen matrix [159], which further decreases the bone’s capacity to distribute loads effectively. It should be noted that mineralization dynamics and cellular functions—particularly the activity of osteoblasts and osteoclasts during remodeling—play integral roles. These interconnected processes together shape the mechanical integrity of aging bone.

The mineral-to-collagen ratio is essential for maintaining bone strength, and this balance may shift with remodeling and aging [158,160]. A study on bovine femoral bones identified an optimal inorganic-to-organic ratio for maximizing bone mass, Young’s modulus, yield stress, and ultimate stress [161]. In healthy young bone, this ratio is tightly regulated. However, age-related changes in collagen structure indirectly alter this balance, impairing mineral incorporation. Water, which provides viscoelasticity essential for load distribution and recovery, also declines with age due to degradation of the collagen matrix [162]. As a result, altered mineral-to-collagen ratios and reduced water content lead to increased bone fragility, a hallmark of age-related skeletal weakening [163].

Bone remodeling and aging also alter bone microstructure [164,165]. As the quantity and quality of regenerated collagen decline, bone mass gradually decreases. In cortical bone, reduced collagen leads to increased porosity and microstructural defects, weakening its resistance to bending and torsional forces. In trabecular bone, collagen loss reduces trabecular thickness and connectivity, fragmenting the trabecular network and impairing its ability to absorb and distribute forces evenly. To counteract these age-related changes, the body adapts at the bone level to help maintain strength, such as by expanding the femoral neck with age [166]. However, the effectiveness of these adaptations diminishes as collagen production further declines, ultimately compromising bone strength and resilience.

### 3.3. Impact of Clinical Risk Factors on Bone Composition and Strength

Although clinical risk factors were previously discussed in Section 2.1 in terms of their association with hip fracture risk from a clinical perspective, they are revisited in this section from a biomaterial standpoint to examine how they influence the composition of bone—specifically, the relative proportions and quality of minerals, collagen, and water. All clinical risk factors, including demographic, bone health and medical conditions, lifestyle and nutrition, and environmental factors, influence hip fracture risk by either altering one or more of the bone’s three primary components (inorganic minerals, organic proteins, and water) or by increasing the risk of falls and subsequent fall-induced impact forces. The first three categories affect bone composition and strength, which will be discussed in this section, while environmental factors mainly influence fall risk and will be covered in Section 4.

For the demographic risk factors, Section 3.2 discussed how aging affects bone composition through remodeling; here, we focus on gender differences in bone composition and strength. From a biological standpoint, men and women vary in bone composition due to hormonal influences that impact the ratio of inorganic minerals, organic proteins, and water [167,168,169]. Post-menopausal hormonal changes discussed in Section 2.1 result in measurable reductions in collagen integrity and increased porosity, contributing to decreased bone mechanical strength [169]. Studies have shown that women experience significant changes in bone microarchitecture and density due to these hormonal shifts [170,171]. In contrast, men, with generally higher testosterone levels, tend to have greater bone mass and mineral density, resulting in denser cortical bone with a more stable mineral-to-organic matrix ratio [169]. Research indicates that men maintain a more consistent bone composition throughout life, with higher bone mineral content (BMC) at specific sites like the femoral neck and distal radius. Additionally, the differences in bone quality and fracture risk between genders are influenced by these compositional variations, with men typically having stronger bones due to higher mineral content and better collagen integrity.

Bone health and medical conditions can significantly affect the quantity and quality of the three bone composition components. Biologically, at each specific anatomic site, an optimal ratio of inorganic minerals, organic proteins, and water is essential for maintaining bone strength and resilience. Imbalances in this ratio can lead to various bone diseases. As described in Section 2.1, osteoporosis results in a loss of bone mineral density (BMD), primarily due to a reduced capacity to regenerate high-quality collagen [172]. Osteomalacia results from inadequate mineralization, leading to soft and flexible bones [173]. Osteogenesis imperfecta, a genetic disorder, disrupts collagen quality, reducing bone flexibility and increasing brittleness [174]. Additionally, conditions like Paget’s disease alter the bone remodeling process, resulting in abnormal bone structure [175]. Each of these diseases highlights how deviations from the optimal composition ratio can compromise bone integrity and lead to pathological fragility.

As discussed earlier, diabetes and CKD are major clinical risk factors. Diabetes, especially type 2, often results in higher bone mineral density but poorer bone quality due to collagen glycation and compromised bone microarchitecture, making bones more brittle [176,177]. Mechanistically, they disrupt both mineralization and the collagen architecture, diminishing bone’s structural resilience [170,174,175,176]. These medical conditions ultimately compromise bone strength and resilience by affecting the three compositional components of bone.

Lifestyle and nutrition factors previously identified in Section 2.1 affect bone health and strength also by impacting the quantity and quality of the three compositional components of bone. Protein intake supports collagen synthesis [178]. Lifestyle factors, such as regular weight-bearing exercise, stimulate bone remodeling, supporting mineral density and collagen integrity [179,180]. Conversely, smoking and excessive alcohol consumption weaken collagen quality and decrease bone hydration [179]. Optimal hydration contributes to bone’s viscoelastic properties, allowing it to absorb impact forces effectively [178]. Consequently, poor lifestyle and nutrition choices can lead to imbalanced bone composition, reducing bone strength and increasing fracture risk [180,181].

## 4. Biomechanical Insights into Fall-Induced Impact Force

**Figure 2:**
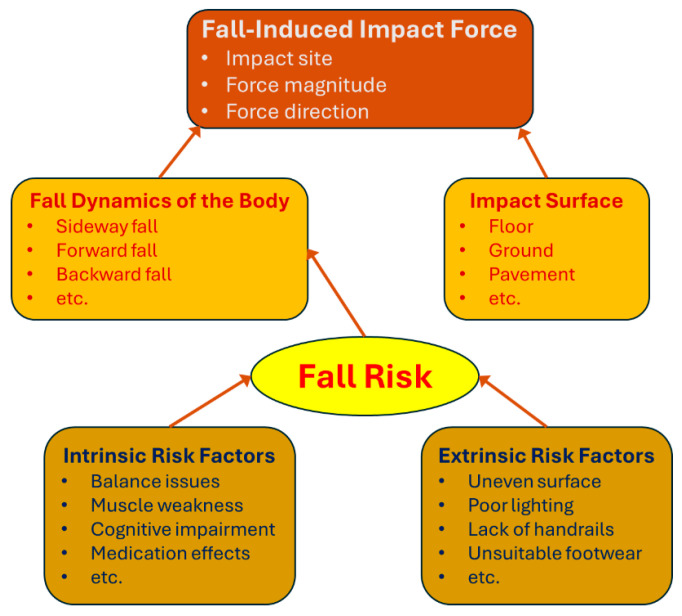
Factors contributing to fall risk and fall-induced impact force.

Fall-induced impact force is the second critical mechanical factor, alongside bone strength, that contributes to hip fracture risk. Falls are the leading cause of hip fractures, especially in older adults, with approximately 95% of hip fractures resulting from fall-related incidents. As illustrated in Figure 2, the triggering mechanisms of a fall and the biomechanical factors that determine the impact force on the body are complex. Due to the combined effect of intrinsic and extrinsic fall risk factors, a fall event leads to an impact force characterized by a specific impact site, magnitude, and direction. These force parameters are governed by the body’s fall dynamics and the compliance of the impact surface, both of which play crucial roles in determining the injury outcome. The complexity and uncertainty of fall-induced impact force are examined in the following subsections, presented in reverse chronological order relative to the sequence of events in a fall.

### 4.1. Biomechanical Mechanisms of Fall-Induced Hip Fractures

Fall-induced impact force is characterized by its impact site, magnitude, and direction, each playing a crucial role in determining hip fracture risk. First, the impact site significantly influences fracture outcomes, as certain areas of the hip are structurally more susceptible to fracture. For instance, an impact at the greater trochanter—a region dominated by cancellous bone with less structural density than cortical bone—greatly increases fracture likelihood. During a fall, the force exerted on the hip can be substantially higher than typical physiological loads, with studies indicating that peak impact forces during a sideways fall from standing height range between approximately 4050 to 6420 N [182], and in some cases reaching as high as 8600 N [183]. In comparison, routine activities subject the hip to forces of only 1800 to 2500 N [184,185]. This sharp increase in force during a fall places considerable stress on the bone, often exceeding its structural capacity and leading to fractures.

In addition to the high magnitude of impact force, as discussed in Section 3.1, bone exhibits anisotropic mechanical properties, meaning its strength and stiffness vary with the direction of the applied force. This directional dependence arises from the organized alignment of collagen fibers and mineral crystals within the bone matrix. For example, cortical bone has greater tensile strength along its longitudinal axis compared to the transverse direction, while trabecular bones are structured to support habitual loading patterns, providing optimal support in specific orientations. Consequently, an impact force misaligned with these natural orientations can exploit the bone’s inherent structural weaknesses, increasing the risk of fracture [186]. This effect is particularly relevant during falls, where the impact direction is often unpredictable and may align with the bone’s weakest axis, thereby heightening the risk of injury.

Therefore, fall-induced impact force can result in fractures even in individuals with strong bones. A hip or other bone fracture caused by a fall does not necessarily indicate low bone strength, which highlights the limitations of using fracture incidence as a validation metric for fracture risk assessment tools like FRAX. This insight may also explain why a significant number of fractures occur in individuals with healthy bone mineral density; in such cases, the alignment and magnitude of fall-induced forces, rather than bone strength alone, are critical factors in determining fracture risk.

### 4.2. Biomechanical Factors Affecting Fall-Induced Impact Force

The impact site, magnitude, and direction of fall-induced impact force are largely influenced by the dynamics of the fall and the compliance properties of the impact surface. Fall dynamics encompass the kinetic and kinematic factors that govern body movement during a fall, including body height, mass, mass distribution, segment length, and other physical parameters [187]. These factors influence the body’s kinetic properties, such as the center of mass and mass moment of inertia. The initial imbalance in posture determines the fall trajectory—whether it results in a forward, sideways, or backward fall. This trajectory dictates the direction and angle of descent, setting the body’s configuration and velocity at the point of impact, and ultimately shaping the characteristics of the impact force.

Muscle strength is pivotal in determining the outcome of a fall. When the body loses balance, adequate muscle strength can facilitate recovery, often preventing a fall from occurring. If balance recovery is not possible, muscle strength can influence body configuration during the descent, such as by lowering the center of mass and reducing the impact force. Research shows that individuals with greater lower limb muscle strength demonstrate improved balance recovery responses, thereby lowering fall risk [188,189]. Studies have also found that older adults with stronger quadriceps muscles are more successful in regaining balance after a perturbation [190], underscoring the importance of muscle strength in fall prevention. Additionally, muscle strength affects body configuration during a fall. Stronger muscles can adjust limb positioning and joint angles, potentially lowering the center of mass and modifying the body’s orientation to reduce injury risk upon impact. For example, individuals with greater hip abductor strength exhibit better control over lateral movements during a fall, decreasing the likelihood of hip fractures [191].

Other physiological parameters, such as body height, mass, and mass distribution, also influence fall outcomes by affecting the body’s kinetic parameters, such as the center of mass and moment of inertia. Taller and heavier individuals may experience greater impact forces due to increased momentum, as momentum is directly proportional to mass [187]. Additionally, body mass distribution impacts how the body rotates and reorients during the fall, influencing both the speed and location of impact with the ground. Kinematic factors, including fall height, significantly affect velocity upon impact. Taller individuals will generally generate higher impact forces due to greater gravitational potential energy at the beginning of the fall, which converts into kinetic energy as they descend. This increased energy contributes to a higher velocity at the point of impact, intensifying the force exerted on the body upon ground contact.

The compliance properties of the impact surface play a crucial role in fall-induced impact force by determining how much energy is absorbed during the collision. Surfaces with higher compliance, such as carpets or padded floors, deform upon impact, absorbing more energy and thereby reducing the force transmitted to the hip or other contact points. In contrast, rigid surfaces like concrete or tile absorb minimal energy, causing the body to bear the majority of the impact force. Studies have shown that thicker soft tissues around the hip can provide cushioning [182], helping to absorb impact energy and further reducing the force that reaches the bone. However, it should be noted that an extremely high body mass index (BMI) may be associated with weaker bone strength, which could counteract some of this protective effect. The compliance of the surface affects not only force magnitude but also force distribution, which can either mitigate or exacerbate injury depending on the interaction between the body and the surface at the point of impact.

Together, muscle strength, kinetic and kinematic parameters establish the body’s velocity, configuration, and energy at impact, ultimately determining the magnitude and orientation of the resulting impact force. Thus, fall dynamics—influenced by muscle strength and the body’s kinetic and kinematic parameters—and the compliance of the impact surface collectively determine the specific impact site, magnitude, and direction of force during a fall. Understanding these biomechanical factors provides valuable insights into why certain falls lead to fractures and emphasizes the importance of targeted interventions, such as strength training and environmental modifications, to reduce fall-related injuries.

While analyses of fall dynamics provide important insights into external risk factors, understanding how these forces translate into internal bone stresses requires individualized biomechanical modeling approaches. To complement descriptive analyses of fall dynamics, patient-specific finite element (FE) simulations offer a powerful tool for predicting internal stress and strain distributions in the femur under fall-related loading conditions. These simulations account for individual anatomical variability and can incorporate realistic loading scenarios. For example, Bini et al. [192] developed subject-specific FE models of the osteoarthritic femoral head that included muscular forces and joint contact mechanics to predict localized stress and strain patterns. This study demonstrates the importance of integrating physiological loading and geometry into fracture risk evaluation and highlights the potential of personalized biomechanical modeling to advance preventive strategies.

### 4.3. Fall Risk and Contributing Factors

Fall risk refers to the probability that a specific individual will experience a fall. Determining fall risk is crucial for hip fracture prevention, as most hip fractures result from falls. By understanding and accurately assessing an individual’s fall risk, targeted preventive measures can be implemented to reduce the likelihood of falls and, consequently, fracture risk. In both clinical and community settings, evaluating fall risk helps identify individuals who would benefit most from interventions such as strength training, balance exercises, and environmental modifications. Given that falls can lead to severe outcomes, especially in older adults with compromised bone strength, assessing and mitigating fall risk is a central component of effective fracture prevention strategies.

Factors affecting fall risk can be divided into intrinsic and extrinsic factors. Intrinsic factors are individual-specific characteristics that influence balance, coordination, and stability. Key intrinsic factors include muscle strength and balance, age and mobility, sensory impairments, cognitive function, and medical conditions [193,194,195,196]. Weak lower body muscles are particularly significant, as they impair balance and make it difficult to recover from a loss of stability. Reduced muscle strength also limits one’s ability to make rapid corrective movements to prevent a fall. Aging is associated with declines in muscle mass, joint flexibility, and proprioception, all of which negatively impact balance and coordination. Limited mobility, often due to joint issues or arthritis, further increases fall risk. Sensory impairments, such as poor vision and hearing, reduce spatial awareness, making it challenging to navigate safely. Cognitive impairments (e.g., dementia or mild cognitive decline) can also increase fall risk by impairing judgment and response time, making individuals less likely to recognize and avoid hazards. Medical conditions, such as diabetes (which can impair nerve sensitivity) and cardiovascular disease (affecting circulation and stability), further compromise balance.

Extrinsic factors are environmental and situational factors that increase fall risk, especially in older adults [84,197,198]. Common extrinsic factors include environmental hazards, footwear, assistive devices, and medications. Environmental hazards—such as uneven surfaces, slippery floors, poor lighting, and cluttered pathways—can significantly increase the chance of falls. Even minor environmental challenges can pose serious risks for older adults. Footwear is another factor; inappropriate shoes, such as high heels or poorly fitting footwear, can undermine stability. In contrast, properly selected assistive devices (e.g., walkers or handrails) can enhance balance and provide support. Finally, certain medications—including sedatives, antidepressants, and blood pressure medications—may cause dizziness or coordination issues, further elevating fall risk.

### 4.4. Challenges in Predicting Fall Risk and Fall-Induced Impact Force

Accurately assessing fall risk and predicting fall-induced impact forces are complex but essential steps in hip fracture prevention. Both processes involve numerous variables, many of which are difficult to measure or predict with precision. Fall risk is influenced by a combination of the intrinsic and extrinsic factors discussed in the previous section. These factors interact in ways that vary widely among individuals, making it challenging to develop standardized assessments that are universally accurate. Additionally, fall risk can fluctuate due to daily variations in health, energy levels, and environmental conditions. For instance, factors like fatigue, recent medication intake, or unexpected obstacles can increase fall risk on any given day, making it difficult to capture these fluctuations with a single assessment.

Some factors affecting fall risk, such as proprioception, cognitive function, and reaction time, are also challenging to quantify objectively. Self-reported information, like physical activity levels or fear of falling, introduces subjectivity and further complicates fall risk assessment. Current tools such as the Timed Up and Go (TUG) test [199] or the Berg Balance Scale [194] offer limited insights and may not consider all relevant factors. These tools are often generalized for population-wide use and may overlook individual-specific risk factors, which can reduce their predictive accuracy.

The challenges in predicting fall-induced impact forces are equally complex. Falls vary considerably in terms of body orientation, velocity, and trajectory, which are influenced by initial posture, body mass distribution, and specific fall conditions. Minor changes in these parameters can lead to substantial differences in impact force, making precise predictions difficult. Additionally, bone strength is anisotropic, meaning it varies depending on the direction of the force applied. An impact aligned with weaker directions in bone strength, especially along a less resilient axis, can increase fracture risk. Accounting for this directional dependence is critical for accurate impact force predictions.

Body–environment interactions further complicate impact force prediction. The compliance of the surface, the cushioning effect of soft tissues, and the body’s orientation at impact all influence the forces experienced during a fall. These variables are difficult to predict without detailed biomechanical models, and variability in environmental factors, such as flooring type and surface rigidity, adds additional complexity. Some individuals may also engage protective responses during a fall, such as extending an arm or adjusting their body position, to reduce the impact force. The effectiveness of these responses depends on muscle strength and reflexes, requiring precise modeling of these individual-specific maneuvers for accurate impact predictions.

Lastly, continuous monitoring of an individual’s activity, balance, and environment holds significant potential for improving the accuracy of fall risk and impact force predictions. While current technologies such as wearable devices show promise, they are not yet widely adopted in clinical practice. Broader implementation of these systems, combined with individualized assessment tools and biomechanical modeling, may enhance the ability to capture fall risk dynamics in real time. Addressing individual variability and the multifactorial nature of falls will be critical to advancing personalized, preventive strategies.

Emerging wearable sensor technologies and real-time monitoring systems are transforming fall risk assessment by enabling continuous, personalized evaluation in real-world settings. Devices such as inertial measurement units (IMUs), pressure-sensitive insoles, and smart garments can capture detailed gait patterns, postural stability, and activity profiles, providing objective data beyond traditional clinical assessments. For instance, Subramaniam et al. [200] reviewed various wearable sensor systems, highlighting their potential in assessing fall risk through parameters like center of pressure trajectory and gait characteristics. The integration of these sensor data into machine learning models has shown promise in enhancing the prediction and prevention of falls. Chen et al. [201] conducted a systematic review, demonstrating that wearable sensor-based technologies, when combined with predictive algorithms, offer accurate and effective fall risk assessments among older adults. These advancements suggest a shift toward dynamic, individualized fall-risk models that can inform timely and targeted interventions.

## 5. Preventative Strategies for Hip Fractures

The best strategy for addressing hip fractures is through prevention, as preventing falls and enhancing bone strength can significantly reduce fracture risk, particularly in older adults. Preventative measures can be broadly classified into passive and proactive strategies. Passive prevention focuses on modifying environments and providing support systems to reduce fall likelihood and minimize impact forces when falls do occur, whereas proactive prevention emphasizes strengthening the individual’s physical and cognitive capabilities to reduce fall risk and improve bone resilience. The following subsections will explore these two approaches in detail, discussing various passive measures, such as environmental modifications and protective equipment, as well as proactive strategies, including cognitive training, muscle strengthening, and overall health improvement.

### 5.1. Passive Preventative Strategies

(1) Environmental Modifications to Reduce the Risk of Falling:

Environmental modifications are a foundational approach to minimizing fall risk, especially for older adults in both home and community settings. Simple adjustments, such as removing loose rugs, securing electrical cords, and keeping pathways clear of clutter, can greatly reduce the likelihood of tripping or stumbling. Adequate lighting, particularly in stairways, hallways, and bathrooms, is essential to improve visibility and reduce hazards. Installing handrails on stairs and grab bars in bathrooms provides stability for those with compromised balance or mobility. Additionally, non-slip flooring or anti-slip mats in high-risk areas, like bathrooms and kitchens, help prevent falls by reducing the likelihood of slipping. In community spaces, thoughtful design considerations, such as ramps, even surfaces, and appropriate railings, can improve accessibility and safety. For older adults, these modifications are particularly valuable in reducing environmental risk factors, making spaces more secure and supportive of their needs.

(2) Modifications to Reduce the Magnitude of Fall-Induced Impact Force:

Beyond reducing fall risk, certain environmental modifications focus on minimizing the force of impact if a fall does occur. Flooring materials with higher compliance, such as carpeted surfaces or cushioned mats, can absorb some of the impact energy, thereby lowering the force transmitted to the body, particularly the hip region. Studies have shown that more compliant surfaces can significantly reduce impact forces and the likelihood of fractures compared to harder surfaces like tile or concrete [202,203]. Additionally, using padded flooring in high-risk areas—such as bathrooms, kitchens, and the area around beds—provides added protection. Soft surfaces can help absorb energy upon impact, although it is important to balance safety with practicality, as overly soft flooring might compromise mobility for some individuals. Nursing homes and elder care facilities, where fall risk is especially high, can consider installing specialized flooring designed to absorb impact energy without significantly affecting ease of movement.

(3) Use of Assistive Devices and Protective Equipment:

Assistive devices and protective equipment can further mitigate the risk and severity of injury in falls. Devices such as walkers, canes, and handrails provide additional support and stability, particularly for those with compromised balance or muscle strength. Walkers and canes distribute weight and improve balance control, allowing individuals to navigate their environments with more confidence. In addition to assistive devices, protective equipment like hip protectors offers another layer of defense against hip fractures [204]. Hip protectors, which are often designed as padded garments worn over the hips, can absorb and disperse impact forces, reducing the force transmitted directly to the bone. These devices are particularly effective in elderly populations or individuals with osteoporosis, for whom a fall is more likely to result in a fracture. While hip protectors and assistive devices do not prevent falls outright, they play a crucial role in lessening fall severity and in protecting the hips and other vulnerable areas during impact.

### 5.2. Proactive Prevention Strategies

(1) Increasing Cognitive Ability to Judge Fall Risk:

Improving cognitive ability to assess fall risk is an essential component of proactive prevention [205], particularly as cognitive decline can impair judgment and decision-making regarding safety. Training programs designed to enhance attention, spatial awareness, and reaction time have shown promise in helping individuals identify and avoid potential hazards [206]. Cognitive exercises—such as memory tasks, problem-solving activities, and spatial orientation exercises—can help maintain and improve brain function related to movement and fall prevention. Additionally, specific interventions like mindfulness training and dual-task exercises, where individuals perform two tasks simultaneously (e.g., walking while solving a puzzle), can improve multitasking and focus, which are crucial for navigating complex or cluttered environments. These cognitive enhancements can foster safer decision-making and increase awareness of risky situations, reducing fall likelihood and fostering greater independence in daily activities.

(2) Improving Balance and Coordination:

Improving balance and coordination is a critical component of fall prevention, as these skills enable individuals to maintain stability during routine movements and respond effectively to unexpected changes in their environment. Balance training specifically targets the neuromuscular system, enhancing an individual’s proprioception (the body’s ability to sense its position in space). Proprioceptive exercises, such as standing on one leg or using balance boards, engage stabilizing muscles and improving reaction times, making it easier to recover from minor stumbles or slips. Activities that emphasize slow, controlled movements, like tai chi and yoga, also improve balance by encouraging focus, posture control, and mindful movement patterns [207,208]. Research has shown that such exercises can significantly reduce fall risk in older adults by reinforcing the body’s ability to make quick, corrective movements and adapt to shifts in weight.

In addition to enhancing balance, coordination exercises help individuals perform movements more smoothly and efficiently, which is especially important when navigating uneven or slippery surfaces. Coordination training often involves complex, multi-joint exercises that require the arms, legs, and core to work together, such as step exercises or ladder drills. These movements strengthen the connections between the brain and muscles, improving both the timing and accuracy of responses. For individuals with existing mobility or balance issues, gradually progressing from simpler exercises to more complex routines is key, allowing the neuromuscular system to adapt and build confidence. By incorporating balance and coordination exercises into a regular routine, individuals can improve stability, reduce fall risk, and maintain independence in their daily activities.

(3) Increasing Muscle Strength:

Although 95% of hip fractures are caused by falls, only a small portion of falls result in fractures, underscoring the essential role of bone strength in fracture prevention [209,210]. Studies indicate a strong association between muscle mass and bone health [211], as muscle exertion places beneficial mechanical stress on bones, promoting bone density and resilience. The relationship between muscle mass and bone strength is well-documented, with research showing that greater muscle mass correlates with increased bone mass [212], partly due to the loading effect that muscles impose on bones, which stimulates bone formation. Collagen, a primary protein in both muscle and bone tissues, is central to maintaining their structural integrity. In bones, collagen fibers form a scaffold that supports mineral deposition, enhancing both strength and flexibility. In muscles, collagen is a key component of the extracellular matrix, influencing muscle elasticity and force transmission. While direct studies on the relationship between muscle collagen and bone collagen quality are limited, the interconnected nature of muscle and bone tissues suggests that factors affecting muscle collagen could also impact bone collagen. For example, conditions that cause muscle atrophy often coincide with bone loss, indicating a systemic relationship between muscle and bone health [213].

Strengthening muscles, especially in the lower body, is critical for improving balance, stability, and the body’s ability to recover from a loss of balance. Resistance training and weight-bearing exercises, such as squats, lunges, and leg presses, target vital muscle groups like the quadriceps, hamstrings, and calf muscles, which are fundamental in supporting posture, controlling movement, and reducing fall risk. In addition, practices like tai chi, yoga, and Pilates enhance muscle strength, flexibility, and balance—key elements in fall prevention. For older adults, regular strength training has been shown to significantly reduce fall risk by enhancing muscular endurance and enabling quick corrective movements during stumbles [214]. By focusing on both strength and balance, these exercises help individuals maintain mobility, increase resilience against falls, and improve overall functional ability.

(4) Improving Overall Health as a Systemic Approach:

Maintaining overall health is crucial for reducing fall risk and enhancing bone resilience, as the body functions as an interconnected system. A balanced diet rich in calcium, vitamin D, and protein supports bone density and muscle health, both essential for preventing fractures. Studies have shown that adequate intake of these nutrients contributes to improved musculoskeletal strength and reduced fracture risk [215,216,217]. Regular physical activity not only strengthens muscles but also supports cardiovascular health, joint flexibility, and respiratory function, all of which contribute to balance and stamina. Multimodal exercise programs—including resistance training, balance exercises, and weight-bearing activities—have been associated with significant reductions in fall and fracture risk among older adults [216,218,219]. Addressing chronic health conditions such as diabetes, hypertension, and arthritis is also essential, as these conditions can impair mobility, sensation, and stability, increasing fall risk. Integrated management strategies that encompass nutrition, physical activity, and medical interventions have been shown to improve musculoskeletal outcomes and reduce the incidence of falls [217,220]. Routine health screenings, medication reviews, and consultations with healthcare providers further assist in managing these conditions and preventing adverse side effects that may elevate fall risk. By adopting a holistic view of health, individuals are better positioned to maintain the strength, coordination, and cognitive function necessary to reduce falls and prevent fractures [219,221].

### 5.3. Perspectives

Looking ahead, the future of hip fracture prevention lies in the convergence of biomechanics, sensor technology, and artificial intelligence. Patient-specific biomechanical models, enhanced by real-time feedback from wearable devices, offer the potential to monitor bone loading conditions and musculoskeletal performance continuously. When coupled with machine learning algorithms trained on multimodal data—including gait patterns, fall history, bone quality, and environmental exposures—these systems can deliver dynamic and individualized risk assessments. Furthermore, the emergence of networked wearable technologies paves the way for closed-loop prevention strategies that adapt interventions in real time, such as triggering balance training exercises or alerting caregivers in response to instability signals. Together, these innovations hold promise for translating biomechanical insights into clinically actionable tools that proactively mitigate fracture risk in aging populations.

## 6. Conclusions

This review offers an integrated perspective on hip fracture risk by synthesizing clinical, biomaterial, and biomechanical insights. It emphasizes how age and disease related changes in bone composition and microstructure contribute to increased mechanical fragility. This review also identifies key limitations in current risk assessment and prevention strategies, underscoring the need for more personalized and multidisciplinary approaches.

Hip fracture is fundamentally a biomechanical failure resulting from the interplay between fall-induced impact forces and the mechanical competence of bone. These two determinants—fall risk and bone strength—form the foundation of fracture risk. While clinical risk factors are diverse, they largely reflect two underlying issues: impaired fall avoidance and compromised bone composition. The latter is governed by the relative proportions and quality of inorganic minerals, organic proteins, and water. From a biomechanical perspective, ideal risk assessment would involve direct in vivo evaluation of bone composition, enabling more accurate insights into mineral density, collagen integrity, and hydration status. However, such techniques remain limited by technical constraints. Moreover, fall-induced impact forces often exceed the physiological load-bearing capacity—particularly when misaligned with the bone’s anisotropic structure—resulting in fractures even in relatively strong bones. This highlights the importance of fall prevention strategies, including balance training and environmental modifications.

Despite progress in clinical diagnostics and preventive interventions, current approaches to hip fracture prevention remain limited. Tools like FRAX provide population-level estimates but lack the resolution needed for individualized risk prediction. Diagnostic imaging methods do not directly assess bone quality in vivo, leaving subtle but important compositional changes undetected. Fall prevention programs are often generalized and fail to address variability in individuals’ biomechanical and neuromuscular profiles. These limitations point to the need for more integrated, individualized, and long-term preventive strategies.

An effective prevention model should combine physical and cognitive interventions. Muscle strengthening improves balance, enhances protective responses during falls, and promotes bone formation. Cognitive training supports fall prevention by improving attention, spatial awareness, and executive function. Together, these strategies address the biomechanical, neuromuscular, and cognitive contributors to hip fracture risk.

In summary, reducing the burden of hip fractures requires an integrated approach that advances risk assessment, enables direct evaluation of bone quality, incorporates fall mitigation strategies, and promotes targeted physical and cognitive training. Future progress will depend on innovations in in vivo bone composition analysis and personalized modeling to support precise, individualized prevention.

## Data Availability

Not applicable.
